# Hexokinase 2 Promotes Cell Proliferation and Tumor Formation through the Wnt/β-catenin Pathway-mediated Cyclin D1/c-myc Upregulation in Epithelial Ovarian Cancer

**DOI:** 10.7150/jca.71894

**Published:** 2022-05-13

**Authors:** Xian Liu, Xiaohang Zuo, Xiaoli Sun, Xueye Tian, Yue Teng

**Affiliations:** 1 Department of Reproductive Medicine, The First Affiliated Hospital of Xi'an Jiaotong University, Xi'an 710061, China; 2 Department of Endocrinology, Xijing 986 Hospital, Fourth Military Medical University, Xi'an 710032, China; 3 Department of Pathology, Baoji Maternal and Child Health Hospital, Baoji 721000, China; 4 Department of Obstetrics and Gynecology, The First Affiliated Hospital of Xi'an Jiaotong University, Xi'an 710061, China

**Keywords:** HK2, CyclinD1, c-myc, ovarian cancer, proliferation

## Abstract

**Background:** HK2 is reported as a key mediator of aerobic glycolysis, associating with the malignant growth in many types of cancers.

**Methods:** In this study, stimulation of HK2 expression was observed in ovarian carcinoma tissues, comparing with the normal ovarian tissues.

**Results:** Both of *in vitro* and *in vivo* experiments demonstrated that HK2 expression promoted the proliferation and tumor formation by accelerating cell cycle progression in ovarian cancer cells. Further research showed that HK2 expression enhanced the activity of Wnt/β-catenin signaling pathway, inducing the protein levels of β-catenin, c-myc and CyclinD1 in HK2 over-expressing OVCA433 and SKOV3 cells. The positive correlation between HK2 and β-catenin, c-myc, CyclinD1 in human ovarian cancer were confirmed from the GEPIA online database. When β-catenin expression was blocked by an inhibitor (XAV939), reduced c-myc and CyclinD1 expression was observed in HK2 over-expressing cells, with inhibited cell growth.

**Conclusion:** Our data demonstrated that hexokinase 2 promotes cell proliferation and tumor formation through the Wnt/β-catenin pathway-mediated CyclinD1/c-myc upregulation in human ovarian cancer.

## Introduction

Epithelial ovarian cancer (EOC) is the leading cause of death from any gynecologic malignancy in the UK and the United States, with less than half of patients living over 5 years from diagnosis 1, 2. This is mainly because the insidious onset and vague presenting symptoms, and over 80% of women present with late-stage disease when first diagnosed 3. Early diagnosis and early treatment is the crucial step to improve patients' treating results and prognosis. Exploration of key markers in EOC onset and progression will definitely contribute to better treatment and prognosis of EOC.

“Warburg Effect” is the oldest known and mysterious hallmark of cancers, refers to the phenomenon that cancer cells preferentially employ a far less efficient way, aerobic glycolysis, to metabolize glucose even in the presence of oxygen 4. Accumulating evidence has suggested this this unique metabolic characteristic is connected with cancerous onset and development 5, 6. Hexokinase 2 (HK2) is a member of the hexokinases family, converts glucose to glucose-6-phosphate, and is the most active and rate-limiting enzyme in glucose metabolism 7. HK2 is found to be abnormally overexpressed in many types of cancer cells and tissues 7. Previous studies have demonstrated that HK2 was involved in multiple cancerous stages, including initiation, maintenance, growth, and metastasis in various cancers 8-11. As for ovarian cancer, HK2 was found to be overexpressed and displayed significantly higher expression in ascites and metastatic foci 12. HK2 was further observed to regulate ovarian cancer metastasis and stemness via FAK/ERK1/2 signaling pathway-mediated MMP9/NANOG/SOX9 expression 12. However, the underlying molecular mechanism of HK2 in regulating cell proliferation and tumor formation in ovarian cancer cells still remains unilluminated.

The Wnt family is a family of proteins that is implicated in many vital cellular activities including organ formation, stem cell renewal, and cell survival 13. Extracellular Wnt can trigger varied intra-cellular signal transduction pathways, like the Wnt/beta-catenin dependent or canonical pathway and the beta-catenin-independent or non-canonical pathway. Wnt/β-Catenin pathway activation has been observed in a variety of malignancies and contributes to tumor recurrence 14-16. Higher Wnt/β-Catenin activity is often observed in EOC. The mechanisms underlying the hyperactivation of the Wnt/β-catenin pathway in EOC and whether it is linked with HK2 interacion are not entirely clear. In our study, exogenous HK2 was stably over expressed in ovarian cancer cells. As shown in this study, HK2 overexpression could activate Wnt/β-catenin pathway by up-regulating β-catenin expression, further promoting cell proliferation and tumor formation by inducing c-myc and Cyclin D1 expression in ovarian cancer cells.

## Materials and Methods

### Cell Lines and Human Tissue Specimens

Human ovarian carcinoma cell lines OVCA433, A2780 and SKOV3 were purchased from the American Type Culture Collection (ATCC, Rockville, MD, USA). High-glucose Dulbecco Modified Eagle Medium (DMEM, Sigma-Aldrich, St Louis, MO, USA) was used to culture OVCA433, A2780 and SKOV3, and 10% fetal bovine serum ((FBS; Hyclone, Thermo Scientific, Waltham, MA, USA) was added in all culture media. The overexpression and shRNA for HK2 was purchased from Gene Pharma (Shanghai, China). Lipofectamine 2000 reagent (Invitrogen, Carlsbad, CA, USA) was used to transfect the pIRES2-AcGFP-HK2 or shRNA vectors into OVCA433, A2780 and SKOV3 cells to generate stably transfected cell lines by treating with G418 (Calbiochem, La Jolla, CA, USA) for 3 weeks.

A total of 21 normal human ovarian (NO), 36 well differentiated epithelial ovarian cancer (WDEOC) and 48 poorly differentiated epithelial ovarian cancer (PDEOC) tissue samples were collected from patients at the First Affiliated Hospital of Xi'an Jiaotong University during 2014 to 2019. These samples were all newly diagnosed, previously untreated. Detailed diagnostic and pathological reports were collected for all patients, and none of them had been previously treated with chemotherapy.

### Immunohistochemistry and Immunocytochemistry

The Immunohistochemistry and Immunocytochemistry used in this study were performed as previously described 17. The staining intensity was scored as: 0 (no staining), 1 (light brown), 2 (brown), 3 (dark brown). The percentage of positive cells was scored as: 0=<5%, 1=5% to 25%, 2=25% to 50%, 3=50% to 75%,4= >75%. The immunohistochemistry (IHC) score = percentage score × intensity score. HK2 staining in tissues was classified into 2 categories (negative and positive expression): a score ≤1 was defined as negative, a score ≥ 2 was defined as strong positive.

The antibodies used were as follows: anti-HK2 (1:200 dilution, sc-374091, Santa Cruz, USA), anti-Ki67 (1:200 dilution, sc-23900, Santa Cruz, USA), anti-Cyclin D1 (1:200 dilution, sc-8396, Santa Cruz, USA), anti-c-myc (1:400 dilution, #18583, Cell Signaling Technology, USA), anti-β-catenin (1:300 dilution, #8480, Cell Signaling Technology, USA).

### Western Blotting

Western blotting analysis used in this study was performed as previously described 18. The antibodies used were as follows: anti-HK2 (1:500 dilution, sc-374091, Santa Cruz, USA), anti-GAPDH (1:100 dilution, sc-47724, Santa Cruz, USA), anti-Cyclin D1 (1:1000 dilution, sc-8396, Santa Cruz, USA), anti-c-myc (1:1000 dilution, #18583, Cell Signaling Technology, USA), anti-β-catenin (1:1000 dilution, #8480, Cell Signaling Technology, USA). The horseradish peroxidase-conjugated anti-rabbit or anti-mouse IgG was purchased from Thermo Fisher Scientific (New York, NY, USA). GAPDH was used as the control and for quantification.

### Cell Growth Assays

Cell proliferation was detected by cell growth curves: 5 × 10^4^ cells were seeded in 6-well plates in triplicate, and cell numbers were counted every 2 days by using hemocytometer.

Cell viability was assessed using 3-(4,5-dimethylthiazole-yl)-2,5-diphenyl tetrazolium bromide (Sigma-Aldrich, St Louis, MO, USA) dye, and the absorbance value at 490 nm was detected by using plate reader.

### Colony Formation Analysis (Soft Agar)

HK2 modified ovarian cancer cells or XAV939 treated cells (5×10^3^ cells) in culture medium containing 10% FBS and 0.35% agarose (low melting, Sigma Chemical Co.) were seeded in 24-well plates pre-coated with 300 mL of 0.7% agarose at 4℃ for 30 min. Cells were incubated at 37℃ for 2 h. Afterward, this culture medium was replaced by culture medium containing 10% SFB, for 24 h at 37 ◦C. Cells were incubated in the culture mediums, control (DMSO) or presence of XAV939 (60 µM) for 3 weeks, with regular change in medium on every other day, at 37℃. Images of three random microscope fields, in duplicate, were captured using an inverted optical microscope. Image J software was used to determine the area of each colony. Only the spheroid-shaped colonies were considered for area calculation. Star-and spheroid shaped colonies above 50 µm were counted. Images are representative of three different experiments.

### Tumor Xenograft Assay

The experimental protocols were evaluated and approved by the Animal Care and Use Committee of the Medical School of Xi'an Jiaotong University and the Ethics Committee of the First Affiliated Hospital of Xi'an Jiaotong University (study reference No.: XJTU1AF2018LSK-245), and all of the animals were raised in a specific pathogen-free (SFP) room, under constant temperature (22-25℃) and humidity (40-50%). Twenty BALB/c-nude mice (female and 4-week-old, purchased from Slac Laboratory Animal Co., Ltd., Shanghai, China) were housed in SPF barrier facilities under a 12 hour light/dark cycle and were randomly divided into four groups (OVCA433-GFP, OVCA433-HK2, SKOV3-GFP and SKOV3-HK2, 5 mice for each group). 1 × 10^5^ HK2 modified ovarian cancer cells and their control cells (100 μl) were subcutaneously injected to the dorsum of each female BALB/c-nude mouse. The mice were killed at the end of the experiment, and all of the tumors derived from HK2 modified ovarian cancer cells were collected and weighed. Following formula was used to measure the tumor volume (V): V =ab^2^/2 (a: length, b: width). The tumors were isolated from mice with a surgical scissor, weighted, and then preserved in 4% PFA at 4℃ for future IHC analysis.

### TOP/FOP Assay

For TOP/FOP assay, the plasmids containing the TOP or FOP firefly luciferase reporters were co-transfected into tumor cells in triplicate using Lipofectamine 2000 (Invitrogen, Carlsbad, CA, USA), with the thymidine kinase promoter Renilla luciferase reporter plasmid (pRL-TK) as an internal control. The activity of both the firefly and Renilla luciferase reporters was determined 48 hours after transfection using the Dual Luciferase Assay kit (Promega, Madison, WI). The specific promoter activity was presented as the relative ratio of firefly luciferase activity to Renilla luciferase activity. The specific activity is shown as the fold change of the experimental group versus the control group.

### Flow Cytometry Analysis

FACS (BD Biosciences, San Jose, CA, USA) was performed for cell cycle analysis, and the data was analyzed by using the Cell-Quest software. 1×10^6^ HK2 modified cells were washed with cold PBS for twice, then fixed in cold ethanol (70%) at 4℃overnight. Next day, cells were washed with cold PBS for twice, then treated with RNaseA (Sigma-Aldrich, St. Louis, MO, USA) and stained with propidium iodide (Sigma-Aldrich, St. Louis, MO, USA).

### Statistical Analysis

All of statistical analysis in this study was performed with Graphpad Prism 8.0 software and SPSS software version 19.0. Two-tailed unpaired Student's t-test was used to determine the statistical significance for 2-group analyses, and presented as mean±SD. Post hoc test was performed for comparison among groups. Chi-square test was used for count data. In all of the tests, statistical significance was defined as *p<0.05, **p<0.01, ***p< 0.001.

## Results

### The expression of HK2 in normal and ovarian cancer lesions

**Figure 1:**
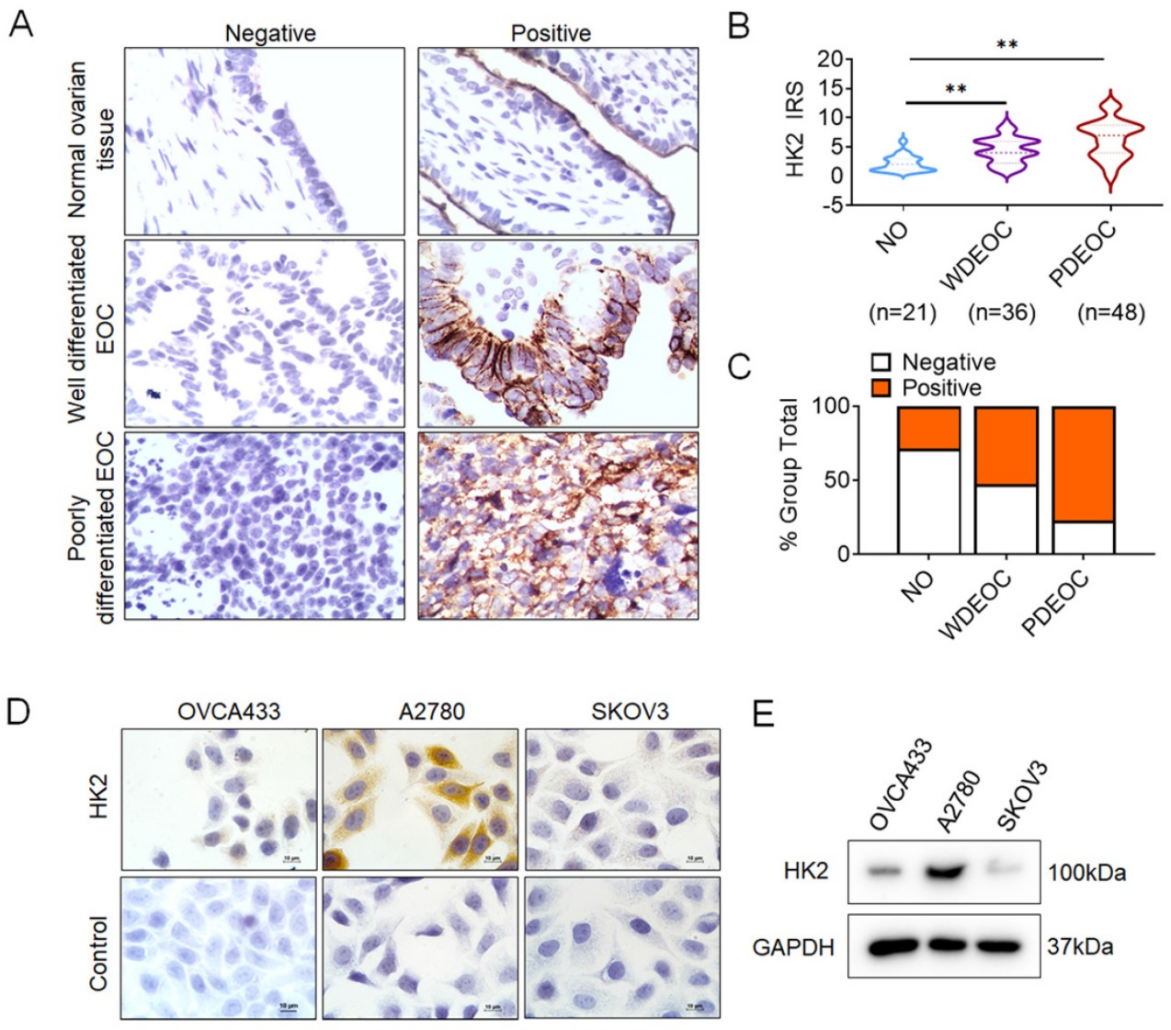
** The expression of HK2 in NO, WDEOC and PDEOC ovarian lesions.** (A) Immunohistochemical (IHC) detection of HK2 in normal ovarian samples (NO), well differentiated epithelial ovarian cancer (WDEOC) and poorly differentiated epithelial ovarian cancer (PDEOC); original magnification, 1000×. (B) The scatter plot shows the immunoreactivity scores (IHC) obtained for the staining of HK2 in normal ovarian samples, well differentiated epithelial ovarian cancer and poorly differentiated epithelial ovarian cancer (points represent the IHC score per specimen, and one-way ANOVA was performed). (C) The negative and positive percentage of HK2 in normal ovarian samples, well differentiated epithelial ovarian cancer and poorly differentiated epithelial ovarian cancer, and the bar chart shows the percentage of each group (21 normal ovarian samples, 36 well differentiated epithelial ovarian cancer and 48 poorly differentiated epithelial ovarian cancer). (D) HK2 expression in human ovarian cancer cell lines (OVCA433, A2780 and SKOV3) was detected by using immunocychemistry. (F) HK2 expression in human ovarian cancer cell lines (OVCA433, A2780 and SKOV3) was detected by using western blotting. Values are shown as the mean±SD, ** *p*<0.01.

To investigate whether HK2 is involved in the development and progression of human ovarian carcinoma, immunohistochemistry (IHC) was used to detect HK2 expression in the normal human ovarian (NO), well differentiated epithelial ovarian cancer (WDEOC) and poorly differentiated epithelial ovarian cancer (PDEOC). Representatively HK2 staining in the NO, WDEOC, and PDEOC lesions was shown in Figure 1A. The average immunoreactivity scores were 2.19 ± 1.30 in NO, 4.42 ± 2.21 in WDEOC, 6.38 ± 3.25 in PDEOC (Figure 1B, NO vs. WDEOC, *p*<0.01; NO vs. PDEOC, *p*<0.01). HK2 protein was localized in the cytoplasm; the positive rate was 28.60% in NO samples (6/21), 52.80% in WDEOC samples (19/36) and 77.00% in PDEOC sample (37/48, Figure 1C and Table. 1). All of these results suggested that HK2 expression was stimulated in ovarian carcinoma tissues and might be involved in the process of ovarian carcinogenesis.

Moreover, HK2 expression was observed in ovarian cancer cell lines (OVCA433, A2780 and SKOV3) using immunocytochemistry (Figures 1D) and western blotting (Figures 1E), and a relatively low expression of HK2 was observed in OVCA433 and SKOV3 cells.

### HK2 promotes cell proliferation of ovarian cancer cells *in vitro*

**Figure 2:**
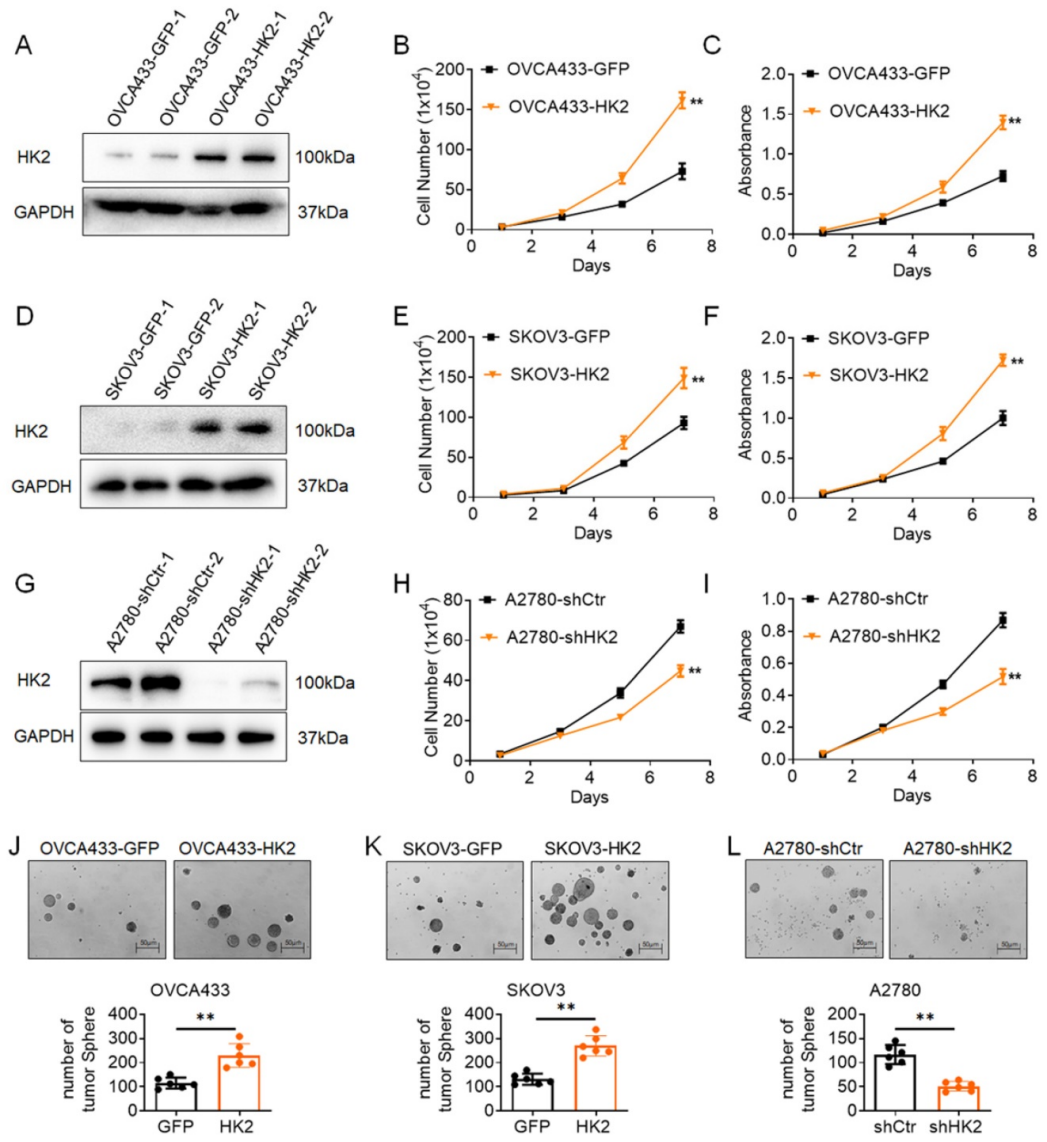
** HK2 promoted cell proliferation of human ovarian cancer cell lines *in vitro*.** The expression of HK2 were identified by western blotting in stably transfected cell lines: (A) OVCA433-GFP and OVCA433-HK2; (D) SKOV3-GFP and SKOV3-HK2; (G) A2780-shCtr and A2780-shHK2. The growth curves were used to detected the cell proliferation in HK2 modified cells: (B) OVCA433-GFP and OVCA433-HK2; (E) SKOV3-GFP and SKOV3-HK2; (H) A2780-shCtr and A2780-shHK2. The MTT assay were used to detected the cell viability in HK2 modified cells: (C) OVCA433-GFP and OVCA433-HK2; (F) SKOV3-GFP and SKOV3-HK2; (I) A2780-shCtr and A2780-shHK2. The soft agar assay for colony formation were used to detected the cell proliferation in HK2 modified cells: (J) OVCA433-GFP and OVCA433-HK2; (K) SKOV3-GFP and SKOV3-HK2; (L) A2780-shCtr and A2780-shHK2. The data were shown as the mean±SD, ** *p*<0.01.

To further investigate the function of HK2 in human ovarian cancer cells, exogenous HK2 was stably overexpressed in OVCA433 (OVCA433-GFP and OVCA433-HK2, Figure 2A) and SKOV3 (SKOV3-GFP and SKOV3-HK2, Figure 2D) cells; conversely, endogenous expression of HK2 was knocked down by stably transfecting shRNA plasmids in A2780 (A2780-shCtr and A2780-shHK2, Figure 2G) cells. According to current research, the abnormal expression of HK2 in cancer cells is linked with tumor initiation and malignant growth in many types of cancers. Therefore, the proliferative activity of HK2 over-expressed and knocked-down cells was detected by using cell growth curves and MTT assays. As shown in Figure 1, the cells grew much faster in HK2 overexpressed OVCA433 (Figure 1B, *p*<0.05) and SKOV3 (Figure 1E, *p*<0.05) cells, comparing with their control cells (OVCA433-GFP and SKOV3-GFP). Moreover, the results from MTT assay also revealed that over-expression of HK2 in OVCA433 (Figure 1C, *p*<0.05) and SKOV3 (Figure 1F, *p*<0.05) enhanced cell viability, comparing with their control cells (OVCA433-GFP and SKOV3-GFP). Conversely, when endogenous expression of HK2 was knocked down in A2870-shHK2 cells, cells exhibited attenuated cell growth (Figure 1H, *p*<0.05) and cell vability (Figure 1I, *p*<0.05), comparing with it control cells (A2780-shCtr).

Moreover, soft agar assay for colony formation was performed to further confirm the biological role of HK2 on regulating the cell proliferation in human ovarian cancer cells. As shown in Figure 1J, the number of formed colonies was much more in HK2 overexpressed OVCA433-HK2 and SKOV3-HK2 cells than their control cells (OVCA433-GFP and SKOV3-GFP). Conversely, when endogenous expression of HK2 was knocked down in A2870-shHK2 cells, the cells exhibited attenuated clone formation ability, comparing with it control cells (A2780-shCtr). These results demonstrated that HK2 promoted cell proliferation in ovarian cancer cells *in vitro*.

### HK2 promotes the tumor growth of ovarian cancer cells *in vivo*

**Figure 3:**
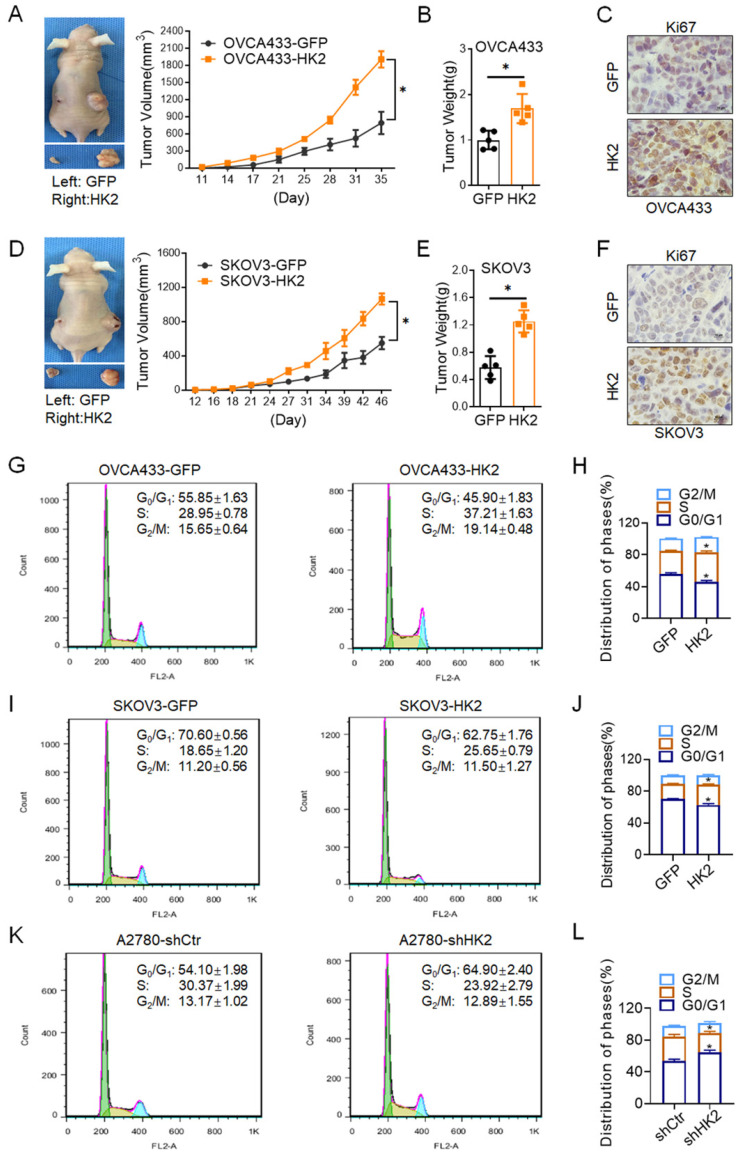
** HK2 promoted ovarian carcinoma tumor growth* in vivo.*** Tumor growth curves were calculated after injection into female nude mice based on monitoring performed every 3 days: (A) OVCA433-GFP and OVCA433-HK2; (D) SKOV3-GFP and SKOV3-HK2. The xenograft tumors that derived from HK2 over-expressed OVCA433 and SKOV3 cells were dissociated and weighed at the end of experiment: (B) OVCA433-GFP and OVCA433-HK2; (E) SKOV3-GFP and SKOV3-HK2. The expression of HK2 and Ki67 in xenograft tumor tissues that derived from HK2 over-expressed OVCA433 and SKOV3 cells were detected by using immunohistochemical. The cell cycle was analyzed in HK2 modified cells by using flow cytometry: (G) OVCA433-GFP and OVCA433-HK2 cells and the quantitative analysis is shown in (H); (I) SKOV3-GFP and SKOV3-HK2 cells and the quantitative analysis is shown in (J); (K) A2780-shCtr and A2780-shHK2 cells and the quantitative analysis is shown in (L). The data were shown as the mean±SD of three independent experiments. * p<0.05, ** p<0.01 vs. control using One-Way ANOVA.

In order to investigate the biological role of HK2 on regulating the proliferative activity *in vivo*, 10^6^ OVCA433-HK2 and SKOV3-HK2 and their control cells were inoculated subcutaneously into female nude mice to identify the role of HK2 in regulating tumor formation *in vivo*. As shown in Figure 3, xenografted tumors derived from OVCA433-HK2 and SKOV3-HK2 grew much larger than those derived from the respective control cells (OVCA433-GFP and SKOV3-GFP, Figures. 3A and 3D, *p*<0.05), and the weight of the tumors formed by the OVCA433-HK2 and SKOV3-HK2 cells was much heavier than that of the tumors formed by their respective control cells (OVCA433-GFP and SKOV3-GFP, Figures. 3B and 3E, *p*<0.05).

Moreover, histoimmunochemistry was used to detected the protein level of Ki67^19^ (a marker for cell prolifration) in OVCA433-GFP, OVCA433-HK2, SKOV3-GFP and SKOV3-HK2 cell derived xenograft tumor tissue. As shown in Figures. 3, comparing with control group, the expression of Ki67 was much higher in OVCA433-HK2 (Figures. 3C, *p*<0.05) and SKOV3-HK2 (Figures. 3F, *p*<0.05) cells derived xenograft tumor tissue, comparing with their control cells (OVCA433-GFP and SKOV3-GFP).

### HK2 accelerate cell cycle progression in ovarian cancer cells

In generally, the abnormal changing of cell proliferation often accompanied with the alteration of cell cycle progression. Thereby, the differences in the distribution of cells in the cell cycle between the HK2-modified cells and their control cells were detected by using fluorescence-activated cell sorting (FACS). As shown in Figure 3G and 3H, HK2 overexpression caused a significantly increased proportion of cells in S phase (37.21 ±1.63 vs 28.95 ±0.78, *p*<0.05) and the G2/M phase (19.14 ±0.48 vs 15.65 ±0.64, *p*<0.05) and a decreased proportion of cells in the G0/G1 phase (45.90±1.83 vs 55.85 ±1.63, *p*<0.05) in OVCA433-HK2 vs OVCA433-GFP cells. Similarly, HK2 overexpression caused a significantly increased proportion of cells in S phase (25.65 ±0.79 vs 18.65 ±1.20, *p*<0.05) and a decreased proportion of cells in the G0/G1 phase (62.75 ±1.76 vs 70.20 ±0.56, *p*<0.05) in SKOV3-HK2 vs SKOV3-GFP cells (Figure 3I and 3J). Conversely, when HK2 was knocked down in A2780 cells, a significantly decreased proportion of cells in S phase (23.92 ±2.79 vs 30.37 ±1.99, *p*<0.05) and an increased proportion of cells in the G0/G1 phase (64.90 ±2.40 vs 54.10 ±1.98, *p*<0.01) in A2870-shHK2 vs A2780-shCrt cells (Figure 3K and 3L).

### HK2 enhanced the activity of Wnt/β-catenin signaling pathway in ovarian cancer cells

**Figure 4:**
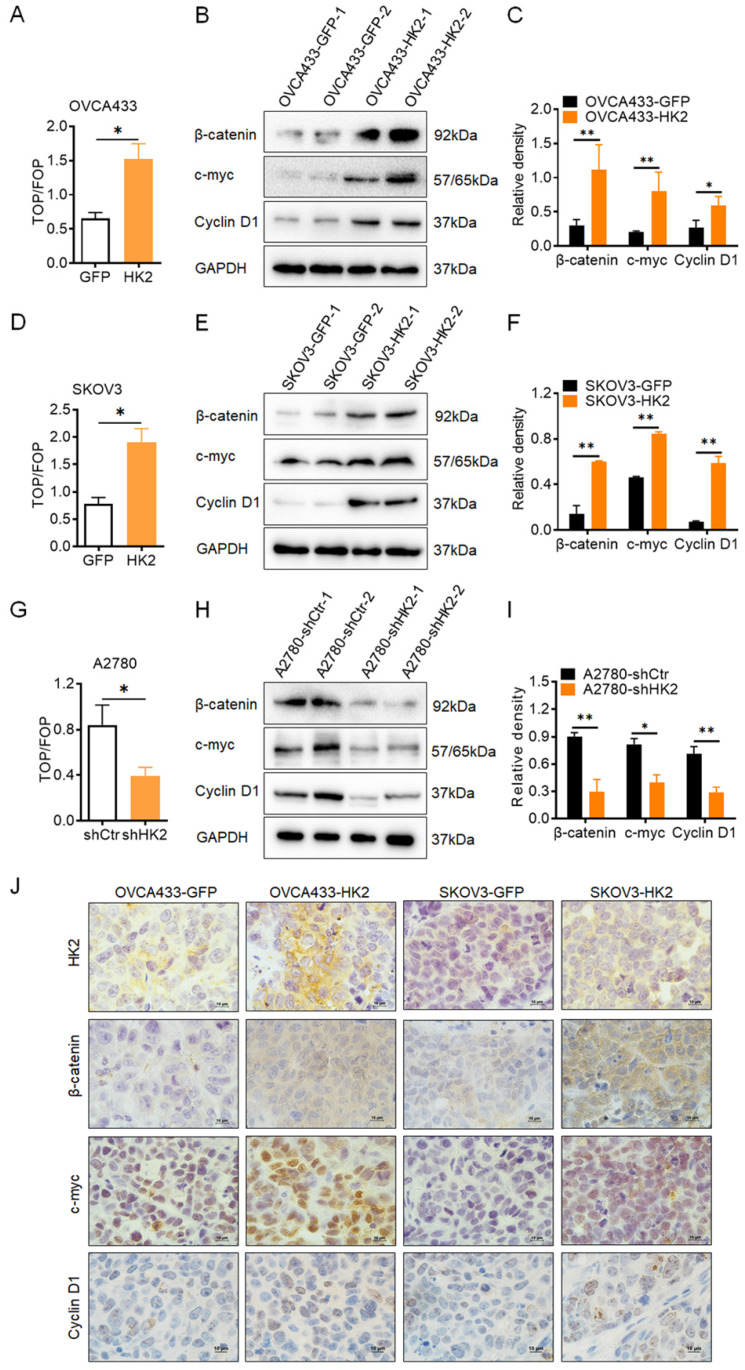
** HK2 altered the Wnt/β-catenin signaling pathway in ovarian cancer cells.** (A) The TOP/FOP-Flash reporter assay was used to identify the activity of the Wnt/β-catenin signaling pathway in HK2-modified ovarian cancer cells: (A) OVCA433-GFP and OVCA433-HK2; (D) SKOV3-GFP and SKOV3-HK2; (G) A2780-shCtr and A2780-shHK2. (B) The expression of β-catenin, c-myc and CyclinD1 was detected by western blotting in OVCA433-GFP and OVCA433-HK2 cells and the quantitative analysis is shown in (C). (E) The expression of β-catenin, c-myc and CyclinD1 was detected by western blotting in SKOV3-GFP and SKOV3-HK2 cells and the quantitative analysis is shown in (F). (H) The expression of β-catenin, c-myc and CyclinD1 was detected by western blotting in A2780-shCtr and A2780-shHK2 cells and the quantitative analysis is shown in (I). (J) The expression of HK2, β-catenin, c-myc and CyclinD1 was detected by immunohistochemical in the xenograft tumors that derived from HK2 over-expressed OVCA433 and SKOV3 cells, original magnification, 1000×. Values are shown as the mean±SD. * *p*<0.05, ** *p*<0.01, *vs.* control using One-Way ANOVA.

In order to determine whether the Wnt/β-catenin signaling pathway could be activated under HK2 expression in ovarian cancer cell lines, the TOP-Flash reporter assay was used to identify the activity of the Wnt/β-catenin signaling pathway in HK2-modified ovarian cancer cells. As shown in Figure 4, in HK2 overexpressed cells, the values of the TOP/FOP assay were much higher in OVCA433-HK2 (Figure 4A) and SKOV3-HK2 (Figure 4D) cells than that in OVCA433-GFP and SKOV3-GFP cells, respectively. Conversely, the values of the TOP/FOP assay were much lower in A2870-shHK2 than that in A2780-shCrt cells (Figure 4G). These data suggested that HK2 expression enhanced the activity of the Wnt/β-catenin signaling pathway in ovarian cancer cells.

Thereby, the protein level of β-catenin, a key factor of Wnt/β-catenin signaling pathway^20^, were detected in HK2-modified ovarian cancer cells. As shown in Figure 4, the protein levels of β-catenin were increased in both HK2 overexpressed OVCA433-HK2 (Figure 4B) and SKOV3-HK2 (Figure 4E) cells, comparing with their control cells, respectively. Conversely, the protein level of β-catenin was much lower in A2870-shHK2 cells than that in A2780-shCrt cells (Figure 4H).

As previous study reported, the canonical Wnt/β-catenin pathway plays a major role in cancer cell proliferation and tumor formation by inducing the gene transactivation of Wnt target genes, such as c-myc and CyclinD1 (the well knowns cell cycle regulatory proteins)^21^, ^22^. Therefore, the expression of c-myc and CyclinD1 were detected in HK2-modified ovarian cancer cells. As shown in Figure 4, the protein levels of c-myc and CyclinD1 were increased in both HK2 overexpressed OVCA433-HK2 (Figure 4B) and SKOV3-HK2 (Figure 4E) cells, comparing with their control cells, respectively. Conversely, the protein levels of c-myc and CyclinD1 were much lower in A2870-shHK2 cells than that in A2780-shCrt cells (Figure 4H).

Furthermore, the histoimmunochemistry was used to confirm the protein level of β-catenin, c-myc and CyclinD1 in OVCA433-GFP, OVCA433-HK2, SKOV3-GFP and SKOV3-HK2 cell derived xenograft tumor tissue. As shown in Figures. 4J, comparing with control group, the expression of β-catenin, c-myc and CyclinD1 was much higher in OVCA433-HK2 and SKOV3-HK2 cells derived xenograft tumor tissue, comparing with their control cells (OVCA433-GFP and SKOV3-GFP). All of these results demonstrated that HK2 altered cell cycle progression by upregulating c-myc and CyclinD1 expression through activating Wnt/β-catenin pathway in ovarian cancer cells, further promoting cell proliferation.

### Blocking the expression of β-catenin in HK2-overexpressing cells inhibits the stimulation of cell proliferation

**Figure 5:**
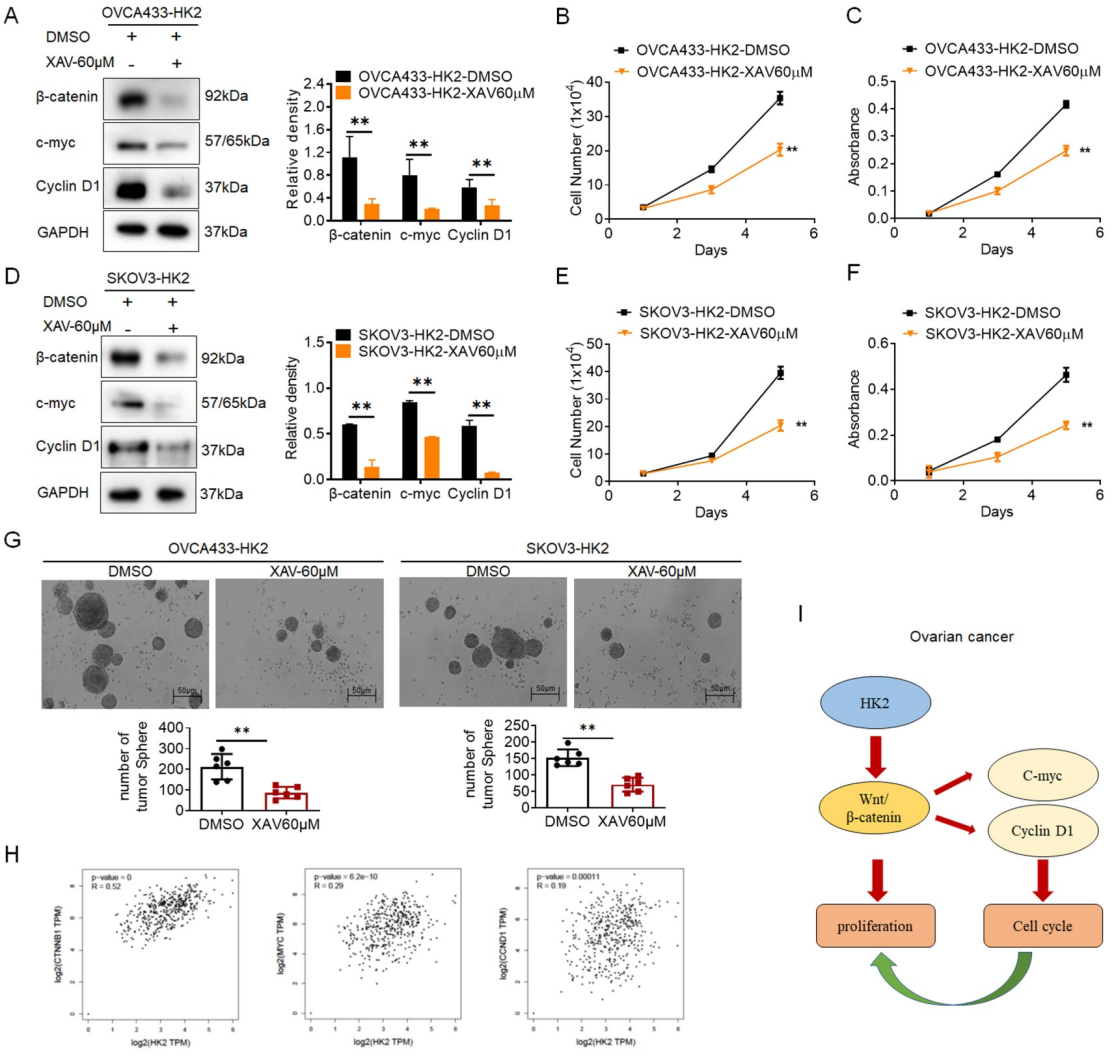
** Blockage of the β-catenin expression in the HK2 over-expressed cells by using XAV939.** The western blotting was used to detected the expression of β-catenin, c-myc and CyclinD1 in XAV939 treated OVCA433-HK2 (A) SKOV3-HK2 (D)cells, and the quantitative analysis is shown. The growth curves (B) and MTT assay (C) was used to detected the proliferation and viability of XAV939 treated OVCA433-HK2 cells. The growth curves (E) and MTT assay (F) was used to detected the proliferation and viability of XAV939 treated SKOV3-HK2. (G) The soft agar assay for colony formation were used to detected the cell proliferation in XAV939 treated OVCA433-HK2 and SKOV3-HK2 cells. (H) The positive correlation between HK2 and β-catenin, c-myc, CyclinD1 expression in ovarian serous cystadenocarcinoma were confirmed from the GEPIA online database. (I) Proposed model of the HK2 promoted cell proliferation and tumor growth in human ovarian cancer. The data were shown as the mean±SD of three independent experiments. * p<0.05, ** p<0.01* vs.* control using One-Way ANOVA.

To further confirm that activated Wnt/β-catenin pathway was responsible for the stimulation of cell proliferation under HK2 overexpressing in ovarian cancer cells, XAV939, an inhibitor of Wnt/β-catenin pathway^23^, was used to block the expression of β-catenin in OVCA433-HK2 and SKOV3-HK2 cells. All cells subjected to XAV939 expressed less β-catenin, c-myc and CyclinD1 (Figures. 5A and 5D, *p*<0.05) and grew much more slowly (Figures. 5B, 5C, 5E and 5F, *p*<0.05) than those cells that were not treated with XAV939. Similarly, the results from soft agar assay for colony formation also revealed that cells subjected to XAV939 formed less colonies than those cells that were not treated with XAV939.

Additionally, the positive correlation between HK2 and β-catenin, c-myc, CyclinD1 in human ovarian cancer were confirmed from the GEPIA online database (Figure 4H, *p*<0.05). All of these results demonstrate that HK2 promotes cell growth and tumor formation by upregulating c-myc and CyclinD1 expression through Wnt/β-catenin pathway in human ovarian cancer cells (Figure 5I).

## Discussion

As reported in previous studies, HK2 expression is frequently upregulated in various human cancers^7^, ^24^, ^25^. Consistently, the increased HK2 expression also observed in human ovarian carcinoma tissues in this study. As shown in Figure 1, the positive expression of HK2 had observed in ovarian cancer cell lines, OVCA433, A2780 and SKOV3, by using immunocytochemistry and western blotting. And the positive rate of HK2 was increased from 28.60% (normal ovarian samples, 6/21) to 52.80% (well differentiated epithelial ovarian cancer samples, 19/36) and 77.00% (37/48) in poorly differentiated epithelial ovarian cancer samples. These results suggesting that HK2 expression was stimulated in ovarian carcinoma tissues and might be involved in the process of ovarian carcinogenesis. HK2 is a key mediator of aerobic glycolysis, and the high rate of aerobic glycolysis is reported to associating with the malignant growth in many types of cancers^25^, ^26^. In this study, both *in vitro* and* in vivo* experiments revealed that the proliferative activity and tumor growth was enhanced in HK2 over-expressed OVCA433 and SKOV3 cells, but attenuated in HK2 knocked-down A2870 cells. Furthermore, cell cycle analysis confirmed the accelerative function of HK2 on promoting cell cycle progression in both HK2 over-expressed OVCA433 and SKOV3 cells. Conversely, cell cycle progression was blocked in HK2 knocked-down A2870 cells. All of these results revealed the promotive effect of HK2 on regulating cell proliferation and tumor formation in ovarian cancer, which consistent with other cancers.

In this study, β-catenin, a key factor involving in the Wnt/β-catenin signaling pathway, was significantly increased in HK2 over-expressed OVCA433 and SKOV3 cells, but decreased in HK2 knocked-down A2870 cells. Moreover, the activation of Wnt/β-catenin signaling pathway was observed in HK2 overexpressed ovarian cancers. CyclinD1 and c-myc were reported as the target genes of Wnt/β-catenin signaling pathway^27^, ^28^, in additionally, both of them also reported as the cell cycle regulatory protein, playing a critical role during cycle phase transition, promoting cell proliferation^29^. Not unexpectedly, c-myc and CyclinD1 also significantly increased in HK2 over-expressed OVCA433 and SKOV3 cells, decreased in HK2 knock-down A2870 cells. When Wnt/β-catenin signaling pathway was blocked by using an Wnt/β-catenin signaling pathway inhibitor (XAV939), induced β-catenin, c-myc and CyclinD1 expression was observed in XAV939-treated HK2 over-expressing cells, and the enhanced cell growth in HK2 over-expressed OVCA433 and SKOV3 cells were also removed by using XAV939. All of these results indicated that this acceleratory effect of HK2 on promoting cell cycle progression in this study likely attributed to the induction of c-myc and CyclinD1 expression through Wnt/β-catenin signaling pathway in ovarian cancer cells. Moreover, the positive correlation between HK2 and β-catenin, c-myc, CyclinD1 in human ovarian cancer were confirmed from the GEPIA online database.

## Conclusion

In conclusion, this study demonstrated that HK2 could promote cell proliferation and tumor formation by inducing c-myc and CyclinD1 expression through Wnt/β-catenin signaling pathway in ovarian cancer cells.

## Figures and Tables

**Table 1:** Correlation between HK2 expression and clinicopathologic characteristics of EOC patients.

Variable	No. patients	HK2 expression		*P*
		Positive	%	
**Age (Years)**				
≤50	46	30	65	*P*>0.05
≥50	38	26	68	
**Differentiation**				
Well	36	19	53	*P*<0.05
Poorly	48	37	77	
**FIGO stage**				
I-II	28	10	36	*P*<0.05
III-IV	56	35	63	

(FIGO, International Federation of Gynecology and Obstetrics stage).
